# Role of Doxycycline as an Osteoarthritis Disease-Modifying Drug

**DOI:** 10.3390/jcm12082927

**Published:** 2023-04-18

**Authors:** Saseendar Shanmugasundaram, Ketansinh Solanki, Samudeeswari Saseendar, Vijay K. Chavada, Riccardo D’Ambrosi

**Affiliations:** 1 Sri Lakshmi Narayana Institute of Medical Sciences, Chennai 605502, India; 2 Bankers Group of Hospital, Vadodara 390007, India; 3 Department of Community Medicine, Indira Gandhi Medical College and Research Institute, Puducherry 605009, India; 4 IRCCS Ospedale Galeazzi—Sant’Ambrogio, 20161 Milan, Italy; 5 Dipartimento di Scienze Biomediche per la Salute, Università degli Studi di Milano, 20133 Milan, Italy

**Keywords:** doxycycline, osteoarthritis, MMPs, knee, hip

## Abstract

Doxycycline is a drug that has been proposed to modify osteoarthritis (OA) progression, in addition to its role as an antibiotic. However, available evidence thus far comprises sporadic reports, with no consensus on its benefits. Hence, this review attempts to analyze the evidence available thus far on the role of doxycycline as a disease-modifying osteoarthritis drug (DMOAD) in knee osteoarthritis. The earliest evidence of doxycycline in OA appeared in 1991 when doxycycline was found to inhibit the type XI collagenolytic activity of extracts from the human osteoarthritic cartilage, and gelatinase and tetracycline were found to inhibit this metalloproteinase activity in articular cartilage in vivo, which could modify cartilage breakdown in osteoarthritis. Apart from the inhibition of cartilage damage by metalloproteinases (MMPs) and other cartilage-related mechanisms, doxycycline also affects the bone and interferes with many enzyme systems. The most significant finding after reviewing various studies was that doxycycline has a definitive role in structural changes in osteoarthritis progression and radiological joint space width, but its role in the improvement of clinical outcomes as a DMOAD has not been established. However, there is much of a gap and lack of evidence in this regard. Doxycycline, as an MMP inhibitor, has theoretical advantages for clinical outcomes, but the present studies reveal only beneficial structural changes in osteoarthritis and very minimal or nonexistent advantages in clinical outcomes. Current evidence does not favor the regular use of doxycycline for the treatment of osteoarthritis as an individual treatment option or in combination with others. However, multicenter large cohort studies are warranted to determine the long-term benefits of doxycycline.

## 1. Introduction

Osteoarthritis is a chronic disease of the joint due to the degeneration of the articular cartilage. One of its characteristics is the progressive loss of matrix constituents, leading to the fibrillation and thinning of the articular cartilage [1].

Inflammation is now a well-acknowledged characteristic of OA, and numerous recent investigations have demonstrated a direct correlation between the presence of synovitis and the development of the condition [2]. Pro-inflammatory (interleukins IL-1b, IL-6, and IL-8) and pro-catabolic mediators through their signaling pathways, as well as the well-known effects of nuclear factor kB (NFkB) and mitogen-activated protein (MAP) kinase signaling responses, are some of the key pathophysiological mechanisms involved in OA [2]. Reprogramming is also referred to as ‘switching’ pathways in transcriptional networks. Inflammatory mediators, mechanical stress, and oxidative stress combine to impair chondrocyte function and viability, making them even more susceptible to the impacts of pro-inflammatory and pro-catabolic mediators. This reprogramming results in early “senescence” and hypertrophic differentiation [2].

Clinical features usually include persistent pain, stiffness, deformity, and functional impairment. The debilitative impact on the individual and the healthcare system is substantial. According to the World Health Organization, 10% of men and 18% of women aged 60 and above have symptoms of OA [3]. However, there are no simple and effective therapies available for the condition. The susceptibility to cartilage injury and resultant OA appears to be even higher, and treatment is more challenging in athletes [4].

Several options have already been established for the management of OA in the knee joint. Hussein et al., in their systematic review of different management options for OA in the knee joint, discussed nonsurgical methods such as (1) lifestyle modifications including regular exercise and weight loss programs for obese individuals, (2) the use of orthoses and footwear, (3) physiotherapy, involving muscle strengthening and aerobic exercises, (4) pharmacotherapy, including nonsteroidal anti-inflammatory drugs, such as selective COX-2 inhibitors. (However, their use has been limited due to potential cardiotoxicity and nephrotoxicity). (5) Several intra-articular preparations were also considered, such as hyaluronic acid (HA), platelet-rich plasma (PRP), bone marrow aspirate concentrate (BMAC), and stromal vascular fraction (SVF) injections [5]. Newer technologies such as 3D bioprinting and gene therapy, are being tested, although it could be a long time before they can be used cost-effectively in clinical practice [6]. Surgical management options include high tibial osteotomy, and cartilage reconstruction procedures such as osteoarticular transplantation, osteochondral allograft transplantation, and autologous chondrocyte transplantation are also being considered. Proximal fibular osteotomy has gained attention recently, although the indications for these procedures are limited [7,8,9]. Patients with severe osteoarthritis usually undergo joint replacement, the outcomes of which can be limited and the treatment expensive.

However, there is no known disease-modifying osteoarthritis drug (DMOAD) for osteoarthritis, and a search for one that targets both symptoms and cartilage structure has intensified over the last few years. Although several studies investigating candidate DMOADs in clinical trials have been published, no agent has stood the test of time to be called irrefutable, as confirmed by DMOAD, as the results of most studies remain largely ambiguous or difficult to interpret [10]. Doxycycline is one such drug that has been proposed to modify osteoarthritis progression in addition to its role as an antibiotic. However, available evidence thus far comprises sporadic reports, with no consensus on its benefits. Hence, this review attempts to analyze the evidence available thus far on the role of doxycycline as a DMOAD in knee osteoarthritis.

## 2. What Is an Ideal DMOAD? (What Is Expected from a Prospective DMOAD?)

An ideal DMOAD is one with an effect on the joint structure, as well as on the symptoms, a long-term clinical benefit, and good long-term safety. According to Losina et al., DMOAD effectiveness can be measured in terms of both structural effectiveness and pain alleviation [11]. A relative decrease in the likelihood of moving from one K-L grade to the next defines structural efficacy. Patients whose OA progression has been stopped by DMOADs (i.e., those for whom DMOADs show structural efficacy) maintain their existing K-L grade. Individuals whose structural advancement has been halted can also feel less discomfort and have a better quality of life. Researchers assumed that DMOAD-related pain alleviation is limited to participants in whom knee OA progression is suspended to ensure a cautious approach concerning the clinical efficacy of DMOADs [11]. Delaying disease progression in the early stages of the condition decreases the quality-of-life declines linked to advanced OA (K-L grade 3 or 4). Major or mild toxicity-affected subjects experience a reduction in quality of life for the next year and must pay for treatment. There is a very small chance of dying from major toxicity. If DMOADs fail to stall the advancement and that failure is identified, or if substantial toxicity occurs, subjects are removed from DMOADs and proceed to the subsequent treatment in the sequence [11].

Currently, several prospective DMOADs are under investigation, some of which are more advanced in development, as summarized in Table 1. However, there are no comparative human studies on doxycycline and other DMOAD agents or adjuvants or comparisons with surgical treatment.

## 3. About Doxycycline

Doxycycline is a tetracycline antibiotic that has been shown to induce the inhibition of cartilage matrix metalloproteinases (MMPs) and slow down the progression of structural damage to the affected joint [22]. It was hence recommended as a disease-modifying therapy for OA. In patients with knee OA, treatment with oral doxycycline may reduce the rate of joint space narrowing, which is regarded as a proxy for cartilage loss of the knee [23]. There is no clear evidence of how or when doxycycline came to be used for osteoarthritis. The earliest evidence of doxycycline in OA appears to be the study of Yu et al. in 1991, where it was found that the drug inhibited the type XI collagenolytic activity of extracts from human osteoarthritic cartilage and that gelatinase and tetracycline could inhibit this metalloproteinase activity in articular cartilage in vivo, which could modify cartilage breakdown in OA [24].

Doxycycline has a long clinical history in humans and is a widely accessible, reasonably priced, and well-tolerated antibiotic that is included in the tetracycline class of antibiotics. Doxycycline has been successfully employed along with the tetracycline-inducible gene regulatory system for controlled gene therapy applications in the articular joint in addition to being an antibacterial [25]. It is an inhibitor of the matrix metalloproteinase (MMP) that cleaves collagen type IX, which is present in articular cartilage [26].

The zinc-dependent proteinases known as MMPs have slightly different substrate specificities. They work by breaking down ECM components, including collagen and proteoglycans, to maintain the equilibrium of the extracellular matrix (ECM) [27]. Collagen retention in the ECM is increased by MMP downregulation [28]. In cases of arthritis and after cartilage damage, MMP is increased [27,28,29]. As MMP-13 is the most active in the breakdown of collagen-II and has been linked to the development of arthritic illness. The overexpression of MMP-13 results in osteoarthritic changes, whereas MMP-13 deficiency prevents cartilage erosion [30,31].

## 4. Action Mechanism of Doxycycline in OA

Through the suppression of matrix metalloproteinase (MMP), the methods by which doxycycline can affect the progression of osteoarthritis were discovered [24]. By demonstrating that 10–30 μM doxycycline prevented the degradation of human type XI collagen in vitro, Yu et al. proposed that doxycycline affects the course of osteoarthritis [24]. Smith et al. characterized the degree to which doxycycline was able to inhibit collagen breakdown by each enzyme in an acellular experiment, adding to the knowledge of doxycycline’s ability to inhibit MMP-1, 8 and 13. Doxycycline decreased the activity of MMP-8 and MMP-13 by 64% and 77%, respectively, at a concentration of 50 M, compared to just 18% for MMP-1 [32].

As doxycycline can downregulate the degeneration of articular cartilage, Brandt et al., along with others, showed that doxycycline can inhibit connective tissue loss through its effects on MMPs, and also affects other pathways that might be important to cartilage integrity, such as nitric oxide production [33].

The probable mechanisms of doxycycline-induced MMP inhibition were further explored by Shlopov et al. in two experiments. In the initial study, TNF- was used to stimulate isolated human knee osteoarthritic chondrocytes while doxycycline was present. Through a decrease in MMP-1, MMP-8, and MMP-13 production and translation in human chondrocytes, doxycycline decreased collagen breakdown [34]. The second study showed that the in vitro treatment of human osteoarthritic chondrocytes with doxycycline dramatically boosted the production of TGF-, while lowering the translation of IL-1, IL-1, and IL-6, in addition to suppressing MMP-1 and MMP-13 [35].

By monitoring gelatinase and collagenase activity in the femoral heads of patients undergoing a total hip arthroplasty for OA, Smith et al. proved doxycycline’s capacity to suppress MMPs. Patients received either oral doxycycline for five days before surgery, in a single dosage three days prior to surgery, or not at all. Samples of the femoral head from patients who took doxycycline for five days showed noticeably decreased collagenase and gelatinase activity [26].

## 5. Effects of Doxycycline on Clinical Profile of Hip and Knee

A Cochrane review of the effects of doxycycline on OA of the hip and knee joint was first published in 2009 and updated in 2012 by Costa et al., who identified a total of 663 participants in two trials. Clinical outcomes were compared, and at the end of treatment, were similar between the two treatment groups with an effect size of −0.05 (a 95% confidence interval, corresponding to a difference in pain scores between the doxycycline and control of −0.1 cm on a 10 cm visual analog scale or 32% versus a 29% improvement from the baseline). The difference between doxycycline and the control on the Western Ontario and McMaster Universities Arthritis Index (WOMAC) disability subscale, which has a range of 0 to 10, was −0.2, representing 24% against a 21% improvement. The effect size for the function was −0.07. Doxycycline showed a difference in the minimum joint space narrowing changes that were measured in one experiment; this difference had a small effect size of −0.23 standard deviation units. The authors concluded that the low-to-non-existent symptomatic benefit of doxycycline outweighed the safety issues and had a slight advantage in terms of joint space narrowing [36,37].

Doxycycline is not effective in lowering symptoms in knee OA patients over a 24-week research period, according to the triple-blinded placebo-controlled study by Snijder et al., which had 232 patients. However, it is linked to an increased risk of side effects. Although doxycycline may affect the structure of cells, this action is not associated with a short- or long-term reduction in symptoms [10].

## 6. Radiological Outcomes after Doxycycline of the Hip and Knee

Various studies have investigated the potential role of doxycycline in reducing radiological joint space narrowing due to its inhibitory effects on cartilage degeneration.

In a randomized placebo-controlled double-blind trial, Brandt et al. studied 431 obese females between the ages of 45 and 64. They discovered a small but significant slowing of the rate of progression in joint space narrowing (JSN), a proxy for cartilage loss, in these subjects’ index knees over 30 months. Nonetheless, there was a slight group difference in other clinical outcomes that favored those treated with doxycycline over those treated with a placebo, despite the lack of any substantial effects on pain [33].

Mazucca et al. conducted a similar study to compare quantitative estimates of change in medial joint space width (JSW) with semiquantitative ratings of joint space narrowing (JSN) and an overall OA severity for their sensitivity to detect a difference between treatment groups concerning the rate of progression of knee osteoarthritis in 431 obese women between 45 and 64 years. Here, doxycycline slowed the mean rate of loss of JSW in the medial compartment of the index knee to 33% at 30 months, compared with the placebo [38]. The same authors in another report estimated the extent to which varus malalignment in the knee could diminish the structurally modifying beneficial effects of doxycycline in the OA of the knee joint and concluded that varus alignment negated the slowing of the structural progression of OA by doxycycline [38,39].

## 7. Role of Doxycycline in Osteoarthritis in Joints Other Than the Hip and Knee

Doxycycline has been studied to observe clinical improvements in the hip and knee joints. Two studies tried to establish its role in other small joints; the first by Israel et al. studied the temporomandibular joint (TM joint) and showed positive results manifested by reduced pain symptoms and the increased range of motion at the TM joint [40], while another study by Ma et al. studied the role of doxycycline in the erosive OA of the hand and reported a 70% improvement in symptoms [41]. However, both studies had only a few participants, warranting further blinded and randomized studies.

## 8. Dosing of Doxycycline as a DMOAD

The serum half-life of doxycycline was 16 h [42]. In vitro, 10 mmol/L doxycyclines inhibited cartilage gelatinase activity by 44%, and 30 mmol/L doxycycline inhibited cartilage gelatinase activity by 82%. Hence, in most RCT studies, a single 100-mg dose of doxycycline was used twice daily [26]. This dosing was similar, as it was given as an antibacterial agent orally or intravenously.

## 9. Safety and Efficacy of Doxycycline

Doxycycline is generally well tolerated and has a long track record of relative safety and well-recognized adverse effects as follows in Table 2:

Nonspecific gastrointestinal complaints were the only adverse events that occurred significantly more often in the active treatment group than in the controls, and none of the adverse events were serious in the reviewed studies for the usage of doxycycline as a DMOAD.

Evidence of the MMP-inhibitor syndrome (shoulder periarthritis, Dupuytren’s contracture) was not observed in any of the studies [33].

## 10. Common Adverse Effects

Doxycycline can cause a variety of adverse effects, but significant ones are extremely uncommon [43]. Gastrointestinal adverse effects might cause candidiasis, nausea, vomiting, and diarrhea. Among the more serious side effects are esophagitis and esophageal ulcerations. If consumed before bedtime and with little to no water, Doxycycline is very harmful [6]. There have also been reports of mediastinitis and hiccups [43].

## 11. Rare Adverse Effects

The rare negative effects of doxycycline include eosinophilia, neutropenia, thrombocytopenia, anemia, and hemolytic anemia. All tetracyclines have the potential to cause benign intracranial hypertension, which can include headaches, nausea, vomiting, lethargy, photophobia, diplopia, and papilledema. Nevertheless, these side effects usually disappear when the medication is stopped. It is also well-documented that adults, children, and pregnant women who use the medication in their second and third trimesters can experience photosensitivity responses and tooth discolorations. A case of doxycycline-induced staining on adult teeth was reported by a woman with previously normal teeth who developed a brownish discoloration after taking doxycycline for acne [44].

### Adverse Effects of Two Forms of Doxycycline

Doxycycline hyclate (DH) is a subtype of the antibiotic that comes in two forms: enteric-coated DH pellets that are time-released in the body (Doryx^®^) and DH powder found in tablets (Vibramycin^®^). They were created to lessen the negative effects, particularly gastrointestinal side effects, of doxycycline [45]. To compare the occurrences of gastrointestinal problems in healthy participants administered Doryx and Vibramycin, Berge [45] conducted a study with double-blind, multiple dosages, placebo-controlled investigation. He discovered that compared to Vibramycin, Doryx induced fewer statistically significant episodes of nausea, vomiting, stomach pain, and decreased appetite. Vibramycin induced more statistically significant symptoms than the placebo for each symptom. However, Berge highlighted the absence of any statistically significant difference in the incidence of other gastrointestinal symptoms between Doryx and the placebo. Therefore, he concluded that when gastrointestinal side effects were considered, Doryx was preferable to Vibramycin. Compared to the doxycycline powder found in tablets, DH time-released pellets (Doryx) had a better gastrointestinal side effect profile (Vibramycin). With Vibramycin, gastrointestinal side effects such as nausea, stomach pain, and decreased appetite were more prevalent [45].

## 12. Clinical Adverse Effects

da Costa et al. reported in their study that 20 people out of 100 who took doxycycline experienced at least one side effect (20%), whereas 15 out of 100 who took the placebo experienced at least one side effect (15%). Thus, five more people who took doxycycline experienced side effects than the placebo consumers (absolute difference of 5%) [37].

Snijder et al., in their triple-blinded placebo-controlled study of 232 patients, reported that 56% of the participants reported at least one adverse event. Of the 28 subjects who prematurely ceased to study medication, 24 did so because of adverse events. The only adverse event observed significantly more often in one of the treatment groups was sun sensitivity. The cumulative incidence of upper respiratory tract infections was, however, somewhat lower in doxycycline-treated patients, although this did not reach a significant value. No studies in the literature have compared the adverse events of various DMOADs [10].

## 13. Future Directions

The most significant finding after reviewing the various items in the literature was that doxycycline has a definitive role in structural changes during OA progression and in radiological joint space width (JSW), but its role in the improvement of clinical outcomes has not been established as a DMOAD. However, there are large gaps in our knowledge and a lack of evidence in this regard. Hence, prospective cohort randomized and blinded studies are warranted to elucidate the matter further, as no recent studies are available.

No comparative human studies are available where the role of doxycycline as a DMOAD is discussed with other available agents or other commonly used intra-articular preparations.

There are no studies in the literature where doxycycline is used as an adjuvant in surgical treatment for OA.

## 14. Conclusions

Doxycycline, a disease-modifying osteoarthritis drug, has theoretical advantages for clinical outcomes, but present studies reveal only beneficial structural changes in osteoarthritis and very minimal or nonexistent advantages in clinical outcomes. Current evidence does not favor the regular use of doxycycline for the treatment of osteoarthritis as an individual treatment option or in combination with other treatment options. However, large, multicenter cohort studies are warranted to determine the long-term benefits of doxycycline.

## Figures and Tables

**Table 1:** Prospective DMOAD studies.

Disease-Modifying Osteoarthritis Agent	Observed Structure Modifying Effects
Glucosamine sulphate	Lower X-ray progression in knee osteoarthtritis (OA) at 3 years [12,13]
Calcitonin	In a 2-year phase 3 trial of knee OA, oral calcitonin modified symptoms and increased cartilage volume but did not affect joint space width (JSW) [14]
Chondroitin sulphate	Lower X-ray progression in knee OA at 2 years [12,13]
Doxycycline	Lower X-ray progression in ipsilateral knee OA at 2.5 years [12,13]
Strontium ranelate	May reduce the X-ray progression of spinal OA [15]
Diacerein	Lower X-ray progression in hip OA at 3 years [13]
Risedronate	No difference in knee OA at 2 years [16]
Zolendronate	Reduction in bone marrow edema (associated with structural changes) and knee pain in 1-year placebo-controlled trial [17]
Bone Morphogenic protein (BMP 7)	Clinical trials on efficacy have commenced but no long-term results available
Hyaluronic acid (HA)	No difference in knee OA at 1 year (less progression in milder disease) [13]
Avocado/soybean unsaponifiables	No difference in hip OA at 2 years (less progression in severe disease) [18]
Fibroblast growth factor (FGF 18)	Can induce chondrogenesis and cartilage repair. It is in phase 2 of clinical trials
Interleukin 1 (IL 1) inhibitors	No improvement in symptoms of human OA studies.
Cathepsin K	A cysteine proteinase–appears to play a role in the pathogenesis of OA. In preclinical models, cathepsin K inhibition reduced evidence of cartilage degradation. It is, therefore, a prospective DMOAD [19,20]
Vitamin D	Several studies are underway on the progression of structural changes in OA
Matrix metalloproteinase (MMP) inhibitors	Failed clinical trial due to musculoskeletal syndrome (MSS). MMP 13 is an important collagenase in OA and its inhibitors are in development as DMOAD
Tissue inhibitors of metalloproteinase	Aggrecan is an important protein found in articular cartilage that is degraded as part of the pathogenesis of OA. Endogenous inhibitors of MMPs include the tissue inhibitor of metalloproteinase TIMP-3, which can inhibit the action of these aggrecanases [21]

**Table 2:** Adverse events of doxycycline.

Adverse Events of Doxycycline as DMOAD [10]
Sun sensitivity
2. Diarrhea
3. Nonspecific gastrointestinal complaints
4. Erythema
5. Nausea
6. Upper respiratory tract infection
7. Arthralgia/myalgia
8. Headache
9. Edema
10. Constipation
11. Mycosis
12. MMP inhibitor syndrome (shoulder periarthritis)

## Data Availability

Not applicable.
